# Evidence of Considerable Shifts in Catch Composition in the Artisanal Spiny Lobster Fishery in Kenya

**DOI:** 10.3390/biology12121477

**Published:** 2023-11-29

**Authors:** Abdirahman J. Kulmiye

**Affiliations:** Department of Zoology, University of Nairobi, P.O. Box 30197, Nairobi 00100, Kenya; akulmiye@kaadinstitute.edu.so

**Keywords:** crustacea, population structure, distribution, exploitation, Kenya

## Abstract

**Simple Summary:**

In Kenya, the artisanal lobster fishery is important socioeconomically for supporting local fishing communities and generating revenue for the government, yet detailed knowledge of many aspects of the fishery is lacking. In this study, the population structure and catch composition of spiny lobsters caught by divers were investigated, and the findings were compared to lobster survey data from the 1970s to identify potential changes in the artisanal landings that may have occurred over time.

**Abstract:**

The artisanal lobster fishery in Kenya is small in world terms but important locally both in terms of supporting local fishing communities and generating revenue for the government. Despite its socioeconomic importance, detailed knowledge of many aspects of the fishery is lacking. The study reported herein aimed to investigate and provide information on the population structure and catch composition of spiny lobsters caught by artisanal fishers off six major landing sites along the coastline. A total of 2711 lobsters representing five palinurid species were collected during the study period (November 2000–March 2001). Among the five species, *Panulirus longipes* dominated the catches in Msambweni (75%) and Shimoni (58%), *P. homarus* in Mambrui (70%) and Kipini (72%), *P. ornatus* in Lamu (49%), and *P. penicillatus* in Kilifi (39%). *P. versicolor* was the rarest species observed in the catches across the six sites. The overall catch consisted of 33% *P. ornatus*, 32% *P. homarus*, 28% *P. longipes*, 6% *P. penicillatus* and 2% *P*. *versicolor*. Sitewise, Lamu contributed 31% of the overall catch, Kipini 23%, Shimoni 20%, Mambrui 13%, Msambweni 7%, and Kilifi 6%. A comparison of the results of this study and lobster abundance data from 1970s surveys revealed considerable shifts in the catch composition of artisanal landings over time. Future work on this fishery should concentrate on the lobster populations in the decades-old marine protected areas to obtain unfished reference data to assess the fishery and establish the underlying cause(s) of the observed shifts in catch composition.

## 1. Introduction

Kenya has both an artisanal and an offshore lobster fishery, of which the artisanal fishery is the most important in terms of production, employment creation, and revenue generation. The offshore production is based on deep-water lobster species (*Metanephrops andamanicus*, *Puerulus angulatus*, Scyllarides) caught as bycatch during semi-industrial prawn trawling off the north coast. The artisanal fishery, on the other hand, exploits five species of shallow-water tropical spiny lobsters belonging to the genus Panulirus, namely, *Panulirus ornatus*, *P. longipes*, *P. homarus*, *P. versicolor* and *P. penicillatus* [1]. 

Artisanal lobster fishing is undertaken along the entire coastline, with the major landing sites being Shimoni, Msambweni, Kilifi, Mambrui, Kipini, and Lamu. Spiny lobsters are harvested year-round, but the peak fishing season occurs during the northeast monsoon (November–April) when weather conditions are favorable. Even though artificial shelters have been shown to attract spiny lobsters at Gazi Bay on the south coast [2], breath-hold diving remains the most popular harvest method. Fishers capture lobsters in shallow waters (<10 m) during the daytime, rarely venturing beyond three nautical miles offshore due to the depth limits of breath-hold diving. Most divers access fishing grounds with dug-out canoes, outriggers, Arab dhows, and other small, non-mechanized crafts [3]. Landed lobsters are either sold to local tourist hotels or exported live to overseas markets, primarily China. 

Although the artisanal spiny lobster production is small in world terms, averaging only 100 metric tons annually, the fishery is socioeconomically important in supporting many fishers and their families and earning the government hard currency through the export of lobsters. Lobster fishing is popular among fisher folks because of the high price lobsters fetch compared to finfish and other crustaceans. Unfortunately, the ever-increasing demand for seafood has led to a tremendous increase in fishing effort in recent years, resulting in overfishing [4]. Declines in catch rates and lobster sizes have been reported for some species [5,6,7]. 

Despite the socioeconomic importance of the resource and the apparent overfishing, the fishery is open access with a few applicable management controls, such as a minimum legal weight of retention (250 g) for all species, prohibitions on retaining berried females, and the use of scuba gear, spears, dynamite, and tangle nets. Four small marine protected areas (MPAs) are also in place where no fishing is permitted. However, these management controls are insufficient to ensure the long-term conservation and sustainable use of lobster resources, even if they are strictly enforced, since they are based mainly on limited scientific information. More comprehensive research is therefore needed to obtain the biological and population parameters in order to formulate stringent, science-based management regulations. Previous studies on Kenyan spiny lobsters have either dealt with a single species or populations in a small geographic area [3,4,6,8,9]. Although both Mutegyera [1] and Mueni et al. [7] focused on the entire coastline, their findings varied substantially, especially regarding the relative abundance of lobster species. This study aimed to investigate and provide information on the species composition, relative abundance, spatial distribution, and population structure of spiny lobsters harvested along the Kenya coast. 

## 2. Materials and Methods

### 2.1. Study Areas

**Figure 1:**
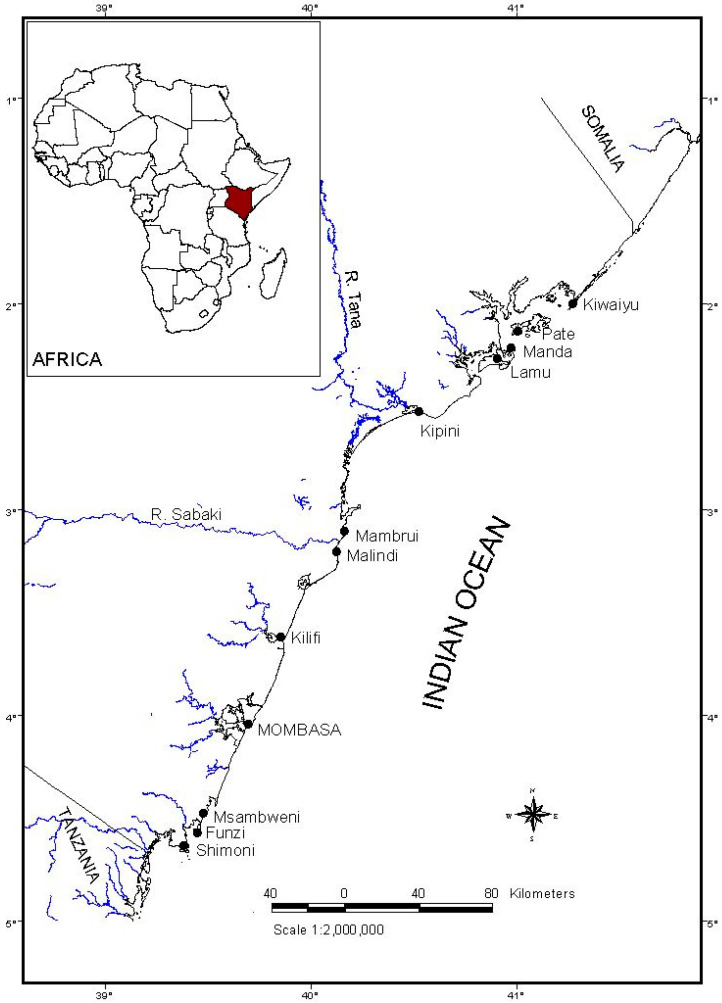
Map of the Kenyan coast showing sampling sites and other lobster landing centers.

The Kenyan coastline is approximately 640 km long, running in a south-westerly direction from the Kenya–Somalia border in the north to the Kenya–Tanzania border in the south (Figure 1). It is fringed by a continuous, well–developed reef that lies between 0.5 and 2 km offshore, except where major rivers (Tana and Sabaki) discharge into the Indian Ocean. The coral reefs, along with seagrasses and mangroves, provide an ideal habitat for spiny lobsters. Generally, the coastline is divided into the north and south coasts, with Mombasa Island serving as the reference point.

For this study, six sampling sites were selected based on their being major lobster landing centers: Shimoni and Msambweni on the south coast, Kilifi, Mambrui, Kipini, and Lamu on the north coast. 

### 2.2. Sample Collection

This study was conducted between November 2000 and March 2001, coinciding with the lobster fishing season. A six-day trip was made to each of the selected six landing sites, where five divers were randomly selected from the respective local fishers and hired to assist with sample collection. In order for their catches to represent the typical landings of a given site, the divers were instructed to fish as they usually would and bring all their lobster catches into a makeshift laboratory at the landing site for analysis. Once brought to the laboratory, lobster samples were identified to the species level, sexed based on external sexual dimorphism, and measured to the nearest 0.1 mm (carapace length, CL) before being returned to the fishers. 

### 2.3. Data Analysis

Lobster CL data were tested for normality (Kolmogorov–Smirnov test) and homogeneity of variance (Levene test) prior to formal analysis. For lobster size comparisons, CL data were grouped by landing site and compared for differences using the Kruskal–Wallis test. When the result was statistically significant (*p* < 0.05), a post hoc test was used to determine which comparisons had significant differences. The sex ratio was determined for each sampling site, and deviation from the expected ratio of 1:1 was tested using the Chi-square (χ^2^) test. *Panulirus penicillatus* and *P. versicolor* were omitted from size and sex composition analyses due to their low proportions or absence from some landing sites. Species composition was calculated based on the number of each species within the catch. Relative abundance was estimated by dividing the number of lobsters in each species by the total number of lobsters in all species. All statistical analyses were performed using SPSS for Windows (version 22.0, IBM, Armonk, NY, USA), and *p*-values lower than 0.05 were considered statistically significant.

### 2.4. Limitations of This Study

The results provided in this study were derived from data collected approximately 22 years ago. Due to the cumulative effects of fishing on exploited stocks, these results may not reflect the current landings of the artisanal lobster fishery in Kenya. However, the findings are important since they can serve as a valuable reference for future assessments of the spiny lobsters in the region. 

## 3. Results

### 3.1. Species Distribution

In this study, five species of spiny lobsters belonging to the genus Panulirus were encountered in the landed catches at the six sampling sites (Table 1). These were *Panulirus ornatus*, *P. longipes*, *P. homarus*, *P. penicillatus*, and *P. versicolor*. *Panulirus ornatus*, *P. longipes* and *P. homarus* were the most widely distributed of the five species, occurring in all six sampling sites. *Panulirus versicolor* was found at four sites, while *P. penicillatus* was encountered at only three sites. Among the six sites, Shimoni, Kilifi, and Lamu had the highest number of spiny lobster species, with all five species present. These were followed by Msambweni and Kipini, with four species each. Mambrui had the least number of species, hosting only three. 

### 3.2. Catch Composition

**Figure 2:**
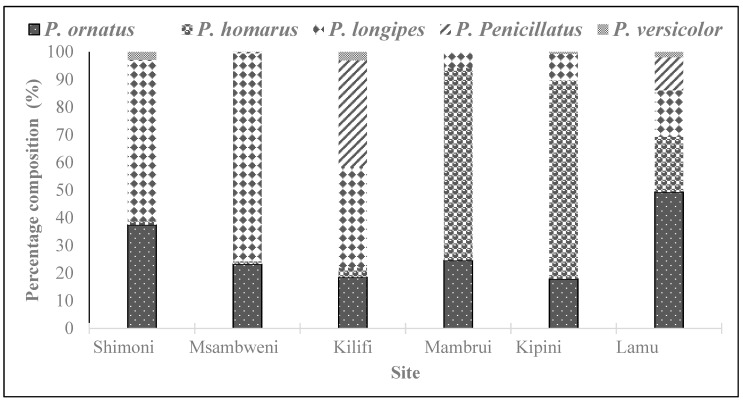
Species composition of spiny lobsters caught off the six sampling sites during the study period (November 2000–March 2001).

**Figure 3:**
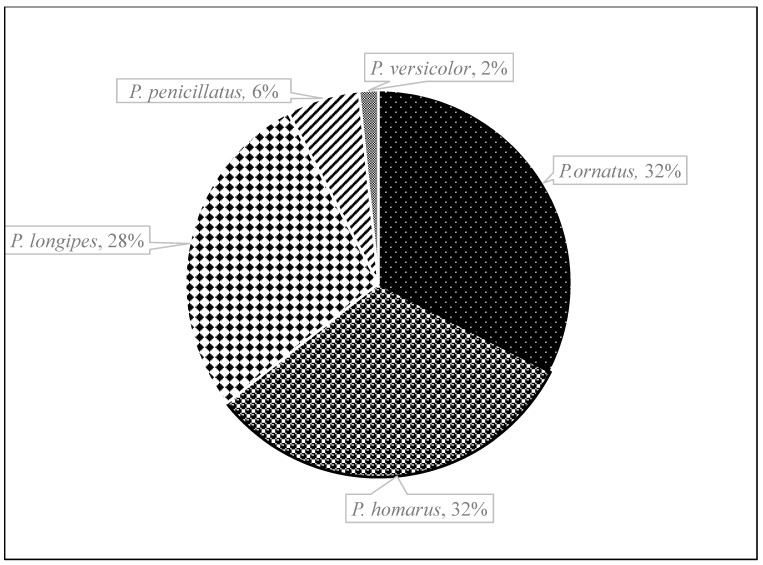
Catch composition of spiny lobsters in the pooled data from all sampling sites.

**Figure 4:**
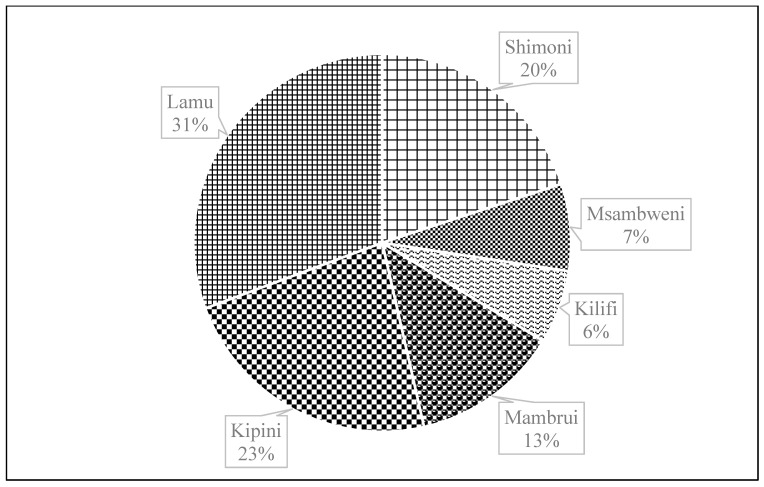
Catch contributions of the respective sampling sites to the pooled spiny lobster data.

A total of 2711 lobsters were collected during the study period, of which 887 belonged to *P. ornatus*, 866 to *P. homarus*, 747 to *P. longipes*, 167 to *P. penicillatus* and 44 to *P. versicolor*. Figure 2 shows the composition of lobster species by landing site. *P. ornatus* was the most abundant species in Lamu, accounting for 49.3% of the sampled catch. It also constituted a substantial proportion of the catches landed at the other five sites. *P. homarus* was most prevalent in the estuarine sites of Mambrui (70%) and Kipini (72%) but least common on the south coast sites of Msambweni and Shimoni, contributing just 1% of the catch. *P. penicillatus* appeared to be the most harvested species in Kilifi. In Msambweni and Shimoni, *P. longipes* accounted for 75% and 58% of the catch, respectively, making it the most important species on the south coast. *P. versicolor* was the rarest species observed in the catches across the sites. Pooling all the catches together (Figure 3), *P. ornatus* had the highest relative abundance (33%), followed by *P. homarus* (32%), *P. longipes* (28%), and *P. penicillatus* (6%), while *P. versicolor* had the lowest relative abundance of just 2%. Sitewise, Lamu contributed most of the pooled catch (31%), followed by Kipini (23%) and Shimoni (20%). The contribution of the other three sites ranged from 6% to 13% (Figure 4). The northern sites of Lamu, Kipini, and Mambrui together accounted for 67% of the lobster landings.

### 3.3. Size and Sex Composition

**Figure 5:**
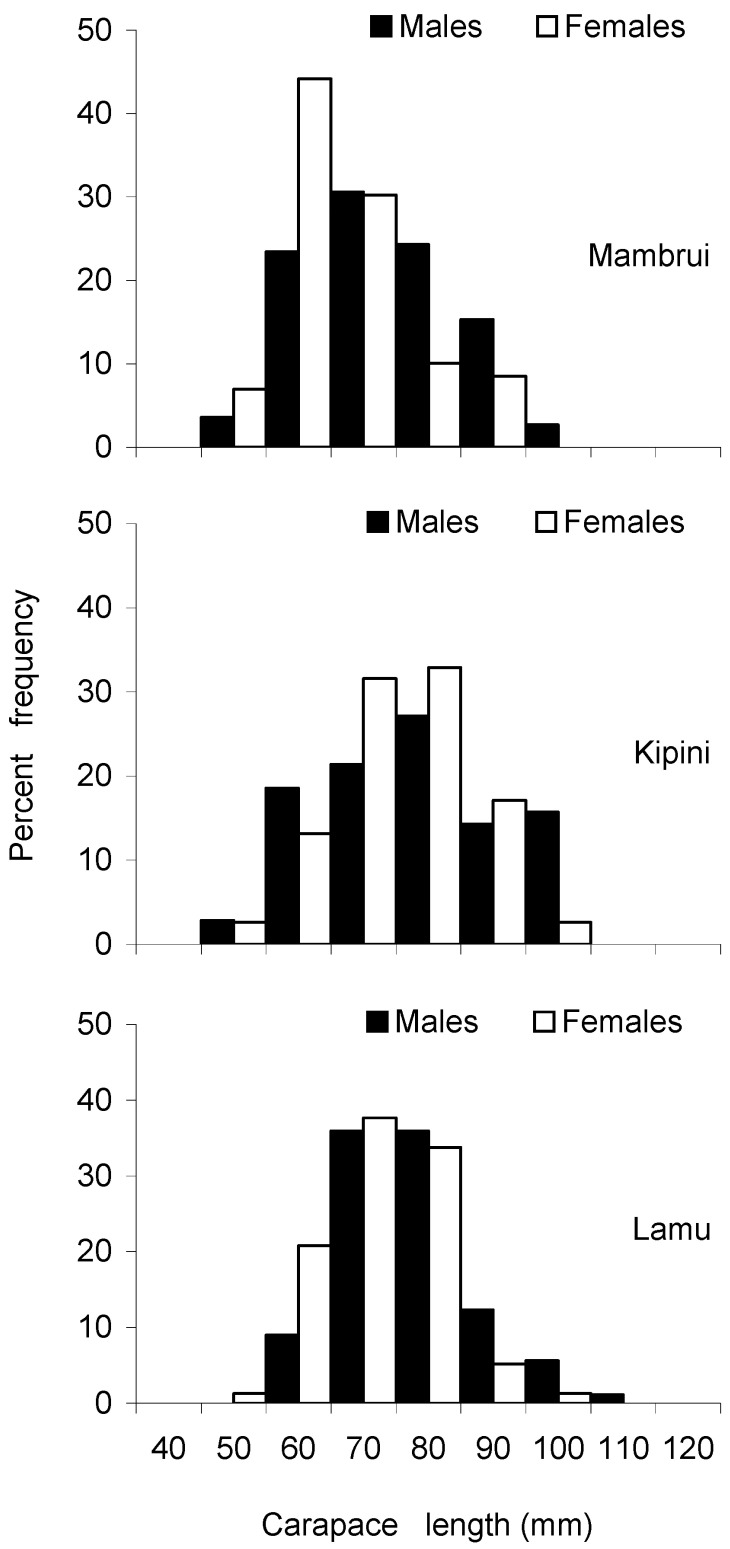
Size frequency distributions of *Panulirus homarus* from three sampling sites during the study period (November 2000–March 2001).

**Figure 6:**
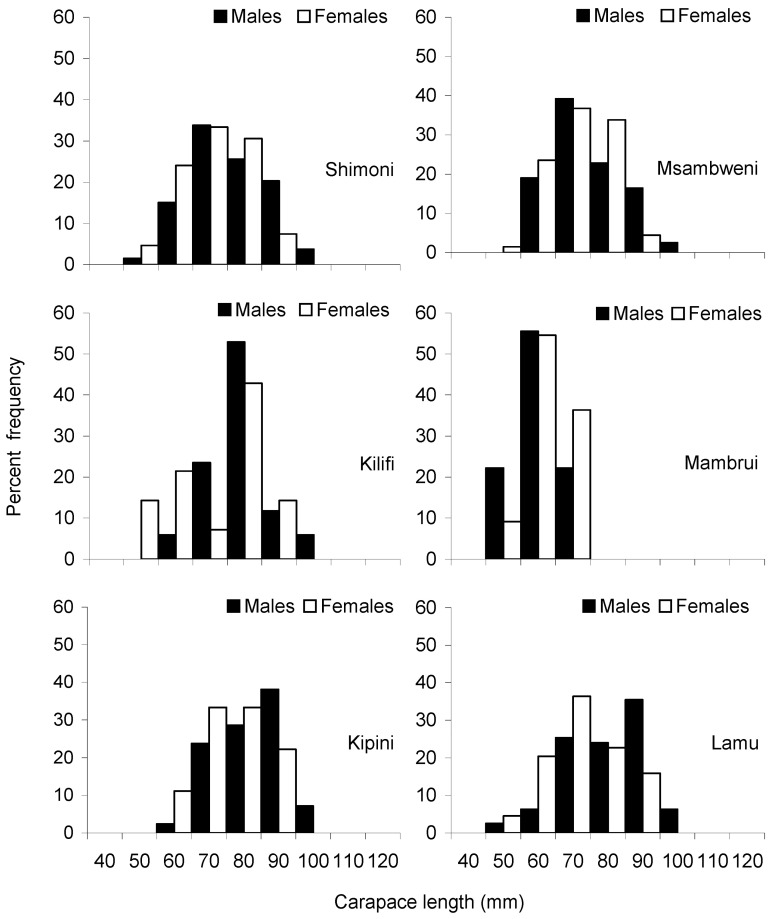
Size frequency distributions of *Panulirus longipes* from six sampling sites during the study period (November 2000–March 2001).

**Figure 7:**
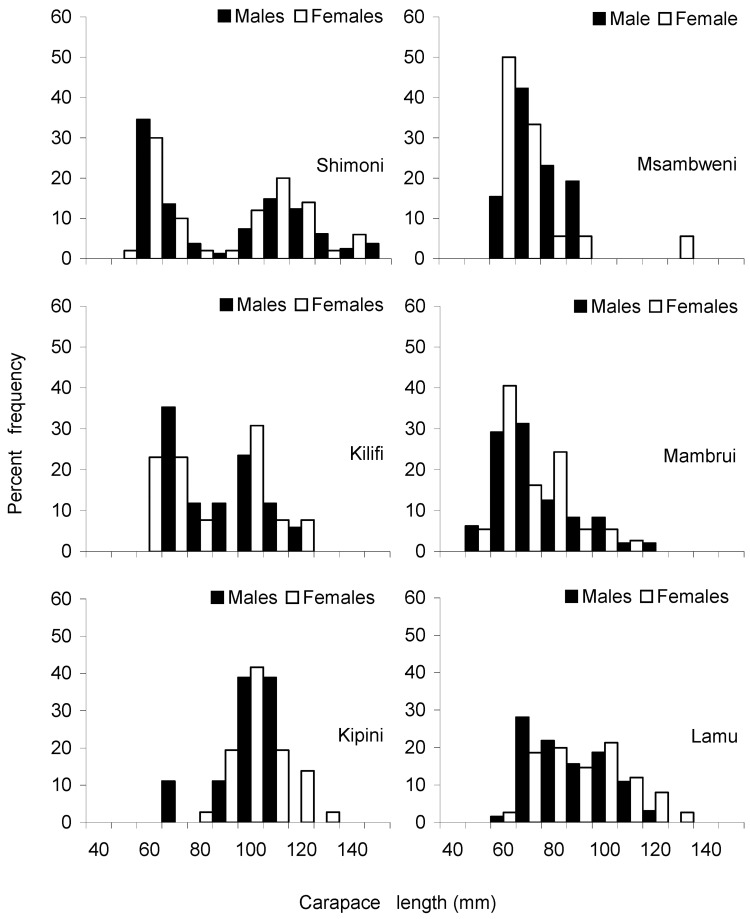
Size frequency distributions of *Panulirus ornatus* from six sampling sites during the study period (November 2000–March 2001).

The descriptive statistics of the carapace length of males and females of the three most abundant species (i.e., *P. ornatus*, *P. homarus* and *P. longipes*) collected from the six sites are presented in Table 2. The overall mean and standard deviation of carapace length were 82 mm ± 22 mm SD for *P. ornatus*, 69 mm ± 12 mm SD for *P. homarus* and 70 mm ± 11 mm SD for *P. longipes*. In terms of the mean size of lobsters by landing sites, lobster samples from Kipini had the largest mean size, while those from Mambrui had the smallest mean size. Carapace length frequency distributions constructed for males and females exhibited a unimodal distribution for all three species over the range of individuals measured (Figure 5, Figure 6 and Figure 7), except for *P. ornatus* males and females from Shimoni and Kilifi and *P. longipes* females from Kilifi, which displayed a bimodal distribution. Analysis of variance (Kruskal–Wallis) test showed significant differences in the lobster size distributions among the landing sites for all three species (Kruskal–Wallis test, *p* < 0.05). Subsequent Dunn’s post hoc tests also revealed significant differences in most paired site comparisons (Table 3).

The sex ratio of *P. ornatus*, *P. homarus* and *P. longipes* determined by site did not depart from the expected male-to-female ratio of 1:1 for all sites (Table 4), except Shimoni and Kipini, where males of *P. ornatus* and *P. longipes* significantly outnumbered the females in the population. In terms of pooled data, *P. ornatus* samples were male-biased, with the sex ratio significantly deviating from a 1:1 ratio. In contrast, male-to-female ratios of *P. homarus* and *P. longipes* in the pooled catch were near parity. 

## 4. Discussion

### 4.1. Species Distribution

In this study, the five species of spiny lobsters previously recorded in Kenya [1,7,8,10] were encountered in the catches, albeit with varying spatial distributions along the coastline. Of the five species, three were found at all six sites, one at four sites, and the remaining at three. Spiny lobster distributions are influenced by various physical factors, including but not limited to seawater quality, wave action, tidal range, and substrate characteristics [11,12,13,14]. While the ecological preferences of the five palinurid lobsters were not investigated in this study, it appears that the nearshore biotopes (coral reefs, mangroves, seagrass beds, and estuaries) are more habitable for *P. ornatus*, *P. homarus*, *P. longipes*, and *P. versicolor* than for *P*. *penicillatus*, given the former four species’ broader distributions. This is entirely in agreement with the views of George [13], who classifies the former four palinurids as continental and coral species that thrive in coastal areas where the climate of adjacent land has a major influence and/or where there are extensive coral formations. In contrast, *P. penicillatus* is found in optimal conditions around oceanic islands away from the influence of terrestrial runoff and rarely occurs in estuarine areas like Mambrui and Kipini, an observation made in this study. 

### 4.2. Catch Composition

The catch composition of the artisanal lobster fishery analysed in this study differed significantly from that of the 1970s. According to Muteryera [1], who conducted lobster assessment surveys between 1975 and 1978, *P. ornatus* often accounted for 80% and, in some cases, 100% of the landings, while *P. homarus* and *P. longipes* each contributed 5% to the catch during that period. The contribution of the remaining two species (*P. penicillatus* and *P. versicolor*) was negligible, rarely exceeding 1% of the total catch. As evidenced by subsequent lobster studies undertaken in 2000 [8], 2000–2001 [present study] and 2013–2014 [7], however, the catch composition appears to have shifted considerably over time in favor of *P. homarus* and *P. longipes*. Both Fielding and Everett [8] and Mueni et al. [7] reported a significant drop in the relative abundance of *P. ornatus* (45%, 55%) and an inverse considerable increase in the proportions of *P. longipes* (20%, 26%) and *P. homarus* (20%, 13%), respectively, compared to the findings of 1970s lobster surveys [1,10]. Mueni et al. [7] did not survey the estuarine sites of Mambrui and Kipini, where *P. homarus* is the most abundant species, which may have underestimated this species’ proportions in the overall catch. In the present study, *P. ornatus* dominated the catch at only one of the six sampling sites, indicating that its historically dominant status as the most harvested species has been reversed along much of the coastline (Table 2). Moreover, its contribution to the overall catch was just 33%, slightly more than that of *P. homarus* (32%) and *P. longipes* (28%), both of which have now become as important in the catch as *P. ornatus.*

Variations in spiny lobster catch composition and abundance across landing sites are determined by various factors, including depth, substratum type, and harvesting method [15]. However, the observed change in the catch composition cannot be explained by these factors alone, given that neither the fishing method nor the lobster fishing grounds have changed over the last seventy years. The increased relative abundance of *P. longipes* and *P*. *homarus* observed both in this research and in the 2000 and 2013/2014 studies suggest that these two species are more resilient to high levels of fishing-induced mortality than *P. ornatus*, probably due to a faster growth rate, higher reproductive output, more successful recruitment, and juvenile survival [8]. In such a scenario, *P. longipes* and *P. homarus* would ultimately outcompete *P. ornatus* under prolonged, intense fishing pressure, provided all other variables remained constant. Although the effects of fishing may partly explain the observed shifts in the catch composition in the artisanal lobster fishery, changes in lobster habitats due to climate-induced coral die-off cannot be ruled out as one of the likely causes of the decreased abundance of *P. ornatus* in recent years. Repeated bleaching and subsequent mortality of shallow-water hard coral may have altered the epi-benthic cover, particularly in terms of shelter and food availability, affecting the recruitment and survival of *P. ornatus* [8]. 

### 4.3. Size Composition

The size composition of lobsters sampled at the six sites varied, with the Mambrui samples containing the smallest specimens among the sites. Variations in the size composition of lobster catches across different geographic locations can be attributed to various factors. These factors include fishing pressure [16,17,18], density-dependent factors [19,20], food availability [21], migration [22], and the combined effects of differences in exploitation levels, oceanographic conditions, and ecological conditions [23]. Although the influence of such factors on the Kenyan spiny lobster populations is unknown and cannot be ruled out, there appears to be a relationship between lobster size composition and the level of fishing intensity at the different sampling sites. Lobsters from less intensely fished areas were considerably larger in size than those from heavily fished grounds. Fishing intensity is higher in areas where both fishing grounds and markets are readily accessible to fishers than in locations where both are remote enough to make lobster fishing less profitable for fishers and dealers. Among the sites, Mambrui and Kipini exemplify the two extremes of the fishing intensity spectrum. 

Mambrui is very close to the popular tourist town of Malindi, with a consistent and substantial demand for lobsters throughout the year. Moreover, the absence of mangroves along this stretch of coastline makes it much easier for fishers to reach the lobster fishing grounds quickly. In contrast, the Kipini lobster fishing grounds lie several kilometers offshore and are further made less accessible by the deltaic nature of the area. The latter site is also located halfway between Malindi and Lamu towns, away from lobster markets, with no reliable road link to either one until recently. 

Because of the artisanal nature of the fishery, the more quantifiable data on fishing effort (i.e., number of lobster boats, fishers, permits, etc.) are not collected systematically across landing sites. The Fisheries Department does not register fishers based on the type of fishing they engage in or their fishing method. Moreover, many fishers do not readily register with the Fisheries Department for fear of paying the required licence fees and other levies. 

Irrational exploitation of spiny lobsters in the form of capture and retention of immature and gravid lobsters has been widespread ever since the commercial harvesting of lobsters started in the mid-1950s. Brusher [10] found that 50% of the fishery comprised undersized lobsters. Mutegyera [1] also noted that 89% of all *P. ornatus* females landed at Kizingitini (Lamu) were below the mean size of the berried females (98.9 mm CL), indicating that a significant percentage of the lobsters were harvested before they had a chance to reproduce. Okechi [24] estimated the mean sizes of male and female *P. ornatus* at first capture at 77.5 mm CL and 67.5 mm CL, respectively, suggesting the capture and retention of immature lobsters. More recently, Mueni et al. [7] reported that *P. ornatus*, *P. homarus* and *P. versicolor* were harvested before they reached the minimum legal weight of retention (250 g) based on the length at first capture they estimated for the three species. In the present study, more than half of all *P. ornatus* collected were smaller than the size (84 mm), at which 50% of females reached size at first maturity [7], confirming the continued harvest of undersized lobsters. During a 2001 survey [5], the most frequent complaints of local fishers along the coastline were the scarcity of lobsters in coastal waters and the declining sizes of lobsters caught. 

## 5. Conclusions

When compared to the data from lobster surveys conducted in the 1970s, the results of this study show a significant change in the catch composition of Kenya’s artisanal lobster fishery over time. In particular, the results show that the traditionally dominant species, *P. ornatus*, has lost its position as the most harvested species along the coastline and now (at the time of this study) ranks marginally higher in terms of abundance than *P. homarus* and *P. longipes*. An interesting question that comes to mind is why only *P. ornatus* has been negatively affected and not the other four species with which it cooccurs in nearshore habitats under the same environmental conditions. Unlike the affected *P. ornatus*, for example, *P. homarus* and *P. longipes* have thrived and become as important in the catch as *P. ornatus* over time, with a combined contribution of 60%, up from 10% in the 1970s. Although several factors are known to influence catch composition and abundance of lobsters, the available data are insufficient to draw any conclusion on the specific factor(s) responsible for the observed shifts in lobster catch composition. Additional research is therefore required to determine the underlying causes of this anomaly, which appears to have varying impacts on the lobster species. In the short term, research on the fishery should focus on the decades-old marine protected areas in order to obtain unfished reference data to empirically assess the status of the fishery. In the long term, however, continuous monitoring of the fishery is required to identify trends and potential issues, such as overfishing or population shifts, for timely regulatory interventions. Alternatively, researchers can use length-based stock assessment models such as length-based spawning potential ratio (LBSPR), length-based indicators (LBI) or length-based Bayesian biomass approach (LBB) to assess the status of the lobster stocks and establish reference points for their management. Length-based models are a viable alternative to conventional catch-based methods for assessing fisheries that lack substantial datasets (i.e., time series of total catch, absolute or relative abundance, fishing effort, life-history parameters, etc.), given their robustness to produce less biased and reliable estimates as well as the simplicity and cost-effectiveness of length data collection. Moreover, the application of length-based models in lobster management can support efforts to assess stock health, evaluate management effectiveness, and explore different length-based management scenarios.

## Figures and Tables

**Table 1:** Distribution of spiny lobster species along the Kenya coast based on samples caught off the six sampling sites during the study period (November 2000–March 2001) *.

Species	Shimoni	Msambweni	Kilifi	Mambrui	Kipini	Lamu
*Panulirus ornatus*	x	x	x	x	x	x
*Panulirus homarus*	x	x	x	x	x	x
*Panulirus longipes*	x	x	x	x	x	x
*Panulirus versicolor*	x	x			x	x
*Panulirus penicillatus*	x		x			x

* Symbol (x) indicates the presence of a species in a particular site.

**Table 2:** Descriptive statistics of the carapace length of male and female spiny lobsters caught off the six sampling sites during the study period (November 2000–March 2001).

		Shimoni		Msambweni		Kilifi		Mambrui		Kipini		Lamu		All
Species		M	F	M	F	M	F	M	F	M	F	M	F	
*Panulirus ornatus*	N	124	79	27	20	18	13	48	37	50	61	215	195	887
	Mean (CL) ± SD	86 ± 31	87 ± 28	69 ± 11	66 ± 17	84 ± 18	79 ± 21	69 ± 16	67 ± 15	95 ± 11	94 ± 10	82 ± 15	87 ± 17	82 ± 22
	Min	55	50	53	53	61	53	42	45	70	79	60	54	42
	Max	155	140	90	125	120	112	116	106	108	127	114	127	155
*Panulirus homarus*	N	4	3	1	1	4	2	111	129	213	232	89	77	866
	Mean (CL) ± SD	72 ± 9	70 ± 11			71 ± 7	64 ± 7	69 ± 12	62 ± 10	74 ± 14	70 ± 11	73 ± 10	68 ± 9	69 ± 12
	Min	61	59	68	57	65	60	45	44	49	49	67	50	44
	Max	82	80			80	69	97	86	100	99	103	93	103
*Panulirus* *longipes*	N	143	170	79	73	33	29	9	11	42	18	75	65	747
	Mean (CL) ± SD	72 ± 11	67 ± 9	70 ± 10	67 ± 8	73 ± 9	67 ± 12	57 ± 7	59 ± 6	77 ± 9	72 ± 10	76 ± 11	68 ± 11	70 ± 11
	Min	45	48	54	50	55	48	45	50	55	51	45	50	45
	Max	97	98	97	89	93	81	68	67	95	87	95	87	97

**Table 3:** Kruskal–Wallis and Dunn’s post hoc test results for the carapace length of spiny lobsters caught off the six sampling sites during the study period (November 2000–March 2001). Statistically significant (*p* < 0.05) values in bold.

*P. ornatus*	Kruskal–Wallis Test			*P. longipes*	Kruskal–Wallis Test			*P. homarus*	Kruskal–Wallis Test		
	H	Degree of Freedom	*p* Value		H	Degree of Freedom	*p* Value		H	Degree of Freedom	*p* Value
Site	89.791	5	**<0.001**	Site	60.357	5	**<0.001**		35.937	4	**<0.001**
Dunn’s post hoc test				Dunn’s post hoc test				Dunn’s post hoc test			
Peer-to-peer comparison	Test static	Standard error	*p* value	Peer-to-peer comparison	Test static	Standard error	*p* value	Peer-to-peer comparison	Test static	Standard error	*p* value
Mambrui vs. Msambweni	−2.409	25.918	0.926	Mambrui vs. Msambweni	−177.724	42.934	**<0.001**	Mambrui vs. Kilifi	57.844	67.464	0.391
Mambrui vs. Kalifi	93.692	29.636	**0.002**	Mambrui vs. Shimoni	−192.952	41.929	**<0.001**	Mambrui vs. Lamu	74.679	16.477	**<0.001**
Mambrui vs. Shimoni	102.079	19.437	**<0.001**	Mambrui vs. Kilifi	227.731	51.687	**<0.001**	Mambrui vs. Kipini	91.055	17.132	**<0.001**
Mambrui vs. Lamu	116.511	19.216	**<0.001**	Mambrui vs. Lamu	258.016	43.450	**<0.001**	Mambrui vs. Shimoni	94.903	62.586	0.129
Mambrui vs. Kipini	195.918	24.286	**<0.001**	Mambrui vs. Kipini	306.742	46.531	**<0.001**	Kilifi vs. Lamu	−16.835	67.830	0.804
Msambweni vs. Kilifi	91.283	33.043	**0.006**	Msambweni vs. Shimoni	−15.227	18.805	0.418	Kilifi vs. Kipini	−33.211	67.992	0.625
Msambweni vs. Shimoni	−99.670	24.317	**<0.001**	Msambweni vs. Kilifi	50.006	35.596	0.160	Kilifi vs. Shimoni	−37.060	90.810	0.683
Msambweni vs. Lamu	114.103	24.140	**<0.001**	Msambweni vs. Lamu	80.292	21.988	**<0.001**	Lamu vs. Kipini	16.376	18.520	0.377
Msambweni vs. Kipini	193.509	28.342	**<0.001**	Msambweni vs. Kipini	129.017	27.581	**<0.001**	Lamu vs. Shimoni	20.224	62.980	0.748
Kilifi vs. Shimoni	−8.387	28.246	0.767	Shimoni vs. Kilifi	34.779	34.378	0.312				
Kilifi vs. Lamu	−22.819	28.095	0.417	Shimoni vs. Lamu	65.064	19.956	**0.001**				
Kilifi vs. Kipini	−102.226	31.778	**0.001**	Shimoni vs. Kipini	113.790	25.990	**<0.001**				
Shimoni vs. Lamu	14.433	16.994	0.396	Kilifi vs. Lamu	−30.285	36.217	0.403				
Shimoni vs. Kipini	93.839	22.568	**<0.001**	Kilifi vs. Kipini	−79.011	39.862	**0.047**				
Lamu vs. Kipini	79.407	22.378	**<0.001**	Lamu vs. Kipini	48.726	28.378	0.086				

**Table 4:** Sex ratios of three spiny lobster species from the six sampling sites during the study period (November 2000–March 2001). Statistically significant (*p* < 0.05) values in bold.

Species		Shimoni	Msambweni	Kilifi	Mambrui	Kipini	Lamu	All
*Panulirus ornatus*	N	203	47	31	85	111	410	887
	Male	124	27	18	48	50	215	482
	Female	79	20	13	37	61	195	405
	Sex ratio (M:F)	1:0.64	1:0.74	1:0.72	1:0.77	1:1.22	1:0.91	1:084
	χ^2^	9.98	1.04	0.81	1.42	1.09	0.98	6.68
	*p*-value	**0.01**	0.31	0.37	0.23	0.30	0.32	**0.01**
*Panulirus homarus*	N	7	2	6	240	445	166	866
	Male	4	1	4	111	213	89	422
	Female	3	1	2	129	232	77	444
	Sex ratio (M:F)	1: 0.75	1:1	1:0.50	1: 1.16	1:1.08	1:0.87	1:1.11
	χ^2^	0.14	0.0	0.67	1.35	0.81	0.87	0.56
	*p*-value	0.71	1.0	0.41	0.21	0.37	0.35	0.46
*Panulirus longipes*	N	313	152	62	20	60	140	747
	Male	143	79	33	11	42	75	381
	Female	170	73	29	9	18	65	366
	Sex ratio (M:F)	1:1.19	1:0.92	1:0.79	1:1.20	1:0.42	1:0.87	1:0.96
	χ^2^	2.33	0.24	0.26	0.20	9.60	0.71	0.58
	*p*-value	0.13	0.63	0.61	0.66	**0.01**	0.40	0.30

## Data Availability

The data presented in this study are available from the author upon request.
